# Brain organoids: from unguided to regionalized to nucleus-specific

**DOI:** 10.1093/lifemedi/lnae014

**Published:** 2024-03-22

**Authors:** Yangfei Xiang, In-Hyun Park

**Affiliations:** School of Life Science and Technology, ShanghaiTech University, Shanghai 201210, China; State Key Laboratory of Advanced Medical Materials and Devices, ShanghaiTech University, Shanghai 201210, China; Shanghai Clinical Research and Trial Center, Shanghai 201210, China; Department of Genetics, Yale Stem Cell Center, Yale Child Study Center, Wu Tsai Institute, Yale School of Medicine, New Haven, CT 06520, United States

Recapitulating subregional identities of the human brain *in vitro* has been challenging. In this commentary, we discuss recent progress in constructing brain organoids with subregional identity.

The brain is an exquisitely complicated organ in the human body. Understanding the human brain has been a task of tremendous challenges for obvious reasons, i.e. technically, and ethically, obtaining data directly from human brain samples is often impractical or not allowed. In addition to model organisms, innovative *in vitro* brain models create pivotal opportunities to explore human brain biology and etiology. One of the emerging *in vitro* models is the brain organoid. Building upon cutting-edge stem cell technologies and growing knowledge in developmental biology, brain organoids, three-dimensional (3D) *in vitro* neural cultures recapitulating essential aspects of the human brain have been developed and rapidly progressed in the past decade.

Efficient differentiation and self-organization of stem cells and progenies in 3D is the key to brain organoid production. In accordance with the “default” model of neural induction and the cell’s self-organization potential, brain organoids can be produced in an unguided manner. For instance, in the cerebral organoid system, minimal induction cues are included, and with the assistance of extracellular matrix, neuroepithelium expansion, and differentiation efficiently occur [1]. The trade-off for this unguided approach is the randomness of lineage differentiation. On the other hand, following neural induction and patterning principles, it is possible to precisely guide stem cell differentiation in 3D, and organoids resembling specific brain regions, such as the cortex, can be generated [2, 3]. Considering the complexity of the human brain, various challenges in brain organoid modeling remain, among which building organoids with finer subregional resolutions (e.g. distinct brain nuclei or brain subregions) largely awaits investigation.

Typical brain structures containing clusters of functional nuclei include the thalamus and hypothalamus. One recent study, reported by Huang et al., pinpointed the arcuate nucleus (ARC) of the hypothalamus [4]. Along with dual SMAD inhibition to induce a neuroectoderm fate, the authors applied activation of SHH signaling (with SHH protein, purmorphamine, and smoothened agonist (SAG) combined) and inhibition of WNT signaling (with IWR-1) starting at the neural induction stage. To promote differentiation and maturation, they cultured organoids in a mouse hypothalamic astrocyte-conditioned medium supplemented with neurotrophic factors. During culture, organoids displayed expressions of genes essential for hypothalamus development, and canonical ARC-specific marker POMC as well as ARC-specific transcription factors. The production of cells related to hypothalamic ARC development was further profiled through single-cell transcriptome analysis of organoids. One of the important demonstrations of organoids’ ARC identity was provided by directly comparing the gene signatures of organoids to those of different adult human hypothalamic nuclei. They also performed single-nucleus RNA-seq of the neonatal human hypothalamus. A machine-learning approach coupled with supervised analysis was applied to predict and validate ARC populations at the single-cell level. Having done that, they compared organoids-derived clusters with different putative human ARC cells, which revealed a significantly higher correlation at the single-cell level in contrast to either thalamic organoids or cortical organoids.

The author then studied Prader-Willi syndrome (PWS), a barely understood neurodevelopmental disorder due to the lack of available human-specific, *in vitro* models. Two induced pluripotent stem cell (iPSC) lines were generated each from two PWS patients carrying either major or minor deletions in the 15q11.2–q13 chromosome region. Compared to organoids generated from healthy donor-derived iPSCs, PWS organoids displayed an altered pattern of development, specifically, with progenitor proliferation increased, neuronal differentiation decreased, and gliogenesis increased. An increase in gliogenesis is in line with observations in postmortem hypothalamus samples of PWS patients. The authors profiled bulk RNA transcriptome of organoids generated from control and PWS iPSCs. Especially, PWS organoids with major genetic deletions showed downregulation of genes related to neural development and upregulation of genes involved in RNA processing, metabolism, cytokine, and stress response, many of which were also observed by others in animal or patient samples. Utilizing the published RNA-seq dataset, they found an overlap of hundreds of downregulated and upregulated genes between gene expression changes in PWS major deletion organoids and PWS patient hypothalamus compared to those of control donors. Thus, there is a possibility that certain alterations in PWS patient hypothalamus may be established early during development.

In the other effort by Kiral et al., a 3D differentiation approach towards a thalamic fate was adopted to identify conditions that favored the production of nucleus-specific lineages [5]. The thalamus can be divided into approximately 60 nuclei, among which the dorsal or caudal region mainly contains excitatory projecting neurons. On the other hand, the thalamic reticular nucleus (TRN) is the primary component of the ventral or rostral thalamus and provides inhibitory feedback to thalamocortical connections. The authors previously developed human thalamic organoids with a regional identity of the dorsal thalamus by dual SMAD inhibition, insulin-induced caudalization, and thalamic patterning (with MEK-ERK inhibition and BMP7 activation). To derive ventral division of the thalamus, they applied activation of SHH signaling to the thalamic organoid protocol. They identified a condition to generate organoids with a ventral thalamic fate. Comparison with distinct human thalamic regions revealed that ventral thalamic organoids recapitulated gene signatures of the human ventral thalamus. Notably, SHH-treated conditions showed similar production of TCF7L2^+^ neurons, suggesting that a shift in the dorsal-ventral axis does not affect the thalamic fate of organoids produced.

The authors discovered a distinct developmental trajectory in ventral thalamic organoids (vThOs) compared to dorsal thalamic organoids (ThOs). Specifically, vThOs mainly produced GABAergic inhibitory neurons (INs) in contrast to ThOs, which mostly generated glutamatergic excitatory neurons. One major cluster of INs was generated mostly from vThOs and expressed canonical TRN markers, referred to as the TRN cluster. Among various validations of inhibitory neurogenesis in vThOs, the author profiled the single-cell transcriptome of organoids at different developmental stages. The ventral identity of vThOs was well-established at the early stage (day 36) and maintained until the late stage (day 112), and INs exhibited maturation during long-term culture. Interestingly, the TRN cluster in vThOs contained four subclusters, mostly absent in ThOs. It was discovered recently that in adult mice, the canonical TRN genes *SPP1* and *ECEL1* mark two clusters of cells that form core- versus shell-like organization. In vThOs, the author did identify SPP1^+^ and ECEL1^+^ subclusters through single-cell transcriptome analysis. Through immunostaining, the separated expression of SPP1 and ECEL1 in vThOs could be validated, albeit no apparent core- versus shell-like structure was observed, possibly because organoids mostly resemble embryonic stages. Functionally, vThOs contained neurons that exhibited a 10-fold increase in burst activity compared to neurons from ThOs, which recapitulates the electrophysiological characteristics of TRN neurons described in animal models. Since TRN sends inhibitory afferents to the dorsal thalamus but not to the cortex, the author investigated the projection patterns of vThO-derived neurons *in vivo*. To this end, they obtained SPP1^+^ TRN cells and SPP1^−^ thalamic neurons, labeled them separately with red or green fluorescence, and transplanted the cells into the thalamus of immune-deficient neonatal mice. Indeed, only SPP1^−^ axonal projections were detected in the cortex.

Thalamic TRN has been involved in neurological disorders such as autism spectrum disorder, schizophrenia, and sleep disturbances, whereas little is known about human TRN, especially in a disease context. The author then investigated two TRN-enriched, disease-related genes as indicated in animal models—*ERBB4* and *PTCHD1*. Suppression of ERBB4 or PTCHD1 expression in vThOs via CRISPR interference revealed a significant decrease in neuronal activity, while cell lineage specification towards the thalamic TRN fate was not affected. Interestingly, the expression of two calcium-activated potassium channels, KCNN2 and KCNN4, was found to increase upon ERBB4 or PTCHD1 knockdown, suggesting a possible common pathway downstream of ERBB4 or PTCHD1 that could regulate KCNN2, KCNN4 and neuronal activity.

**Figure 1.**
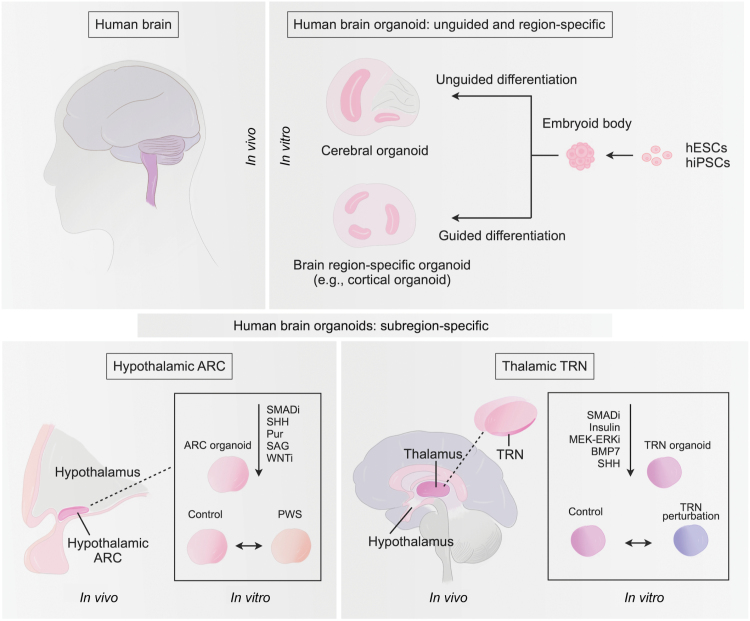
**Current strategies in constructing human brain organoids.** Brain organoids can be generated from human pluripotent stem cells (hESCs and hiPSCs). Cerebral organoids are established through an unguided differentiation approach, whereas distinct brain region-specific organoids, such as cortical organoids, are generated via guided differentiation. Most recently, brain organoids with nucleus-specific identities, such as the human hypothalamic ARC and thalamic TRN, are generated by precisely controlling cell fate in 3D differentiation.

The above two studies developed human brain organoids with subregional resolutions. As proof-of-concept experiments, they took advantage of organoids’ nucleus-specific identities and explored potential pathological mechanisms related to the human ARC or TRN nucleus. Numerous questions remain. Particularly, nucleus-specific organoids are more similar to the developing human brain than the adult brain. Thus, refined cytoarchitectures of adult brain nuclei are challenging to recapitulate. Introducing other components, such as blood vasculatures that would benefit long-term maturation and target brain regions to which the nucleus of interest connects may help to build more advanced nucleus-specific brain organoids. Considering the variety of functional brain nuclei, an equally critical need is to develop brain organoids targeting nuclei involved in human brain diseases, which will allow us to understand human brain development and related diseases at a finer resolution. The endeavors towards building brain organoids with nucleus-level subregional identities will also broaden our knowledge about controlling differentiation in 3D in an ever-precise manner. For disease modeling, the generation of brain region-specific and nucleus-specific organoids will eventually allow a more precise and systematic survey of the human brain, given that neurological disorders may often involve multiple brain regions’ dysfunction, which is challenging to decipher *in vivo*. Overall, from unguided cerebral organoids to various brain region-specific organoids to the latest nucleus-specific organoids (Fig. 1), efforts to advance the technology further have been ongoing for over a decade and will continue. All these efforts, together with other model systems, will undoubtedly deepen our understanding of the human brain and diseases.
